# A typology of uncertainty derived from an analysis of critical incidents in medical residents: A mixed methods study

**DOI:** 10.1186/s12909-015-0459-2

**Published:** 2015-11-04

**Authors:** Alicia Hamui-Sutton, Tania Vives-Varela, Samuel Gutiérrez-Barreto, Iwin Leenen, Melchor Sánchez-Mendiola

**Affiliations:** 1 Faculty of Medicine, Universidad Nacional Autónoma de México, Mexico City, Mexico; 2 Instituto Nacional para la Evaluación de la Educación, Mexico City, Mexico; 3 Unidad de Posgrado, División de Estudios de Posgrado, Ciudad Universitaria Mexico, Edificio G, 2 piso, oficina G226, C.P. 04510, Coyoacán, D.F. Mexico City, Mexico

**Keywords:** Resident education, Medical uncertainty, Critical incidents, Coping strategies, Decision making, Clinical context, Uncertainty typology

## Abstract

**Background:**

Medical uncertainty is inherently related to the practice of the physician and generally affects his or her patient care, job satisfaction, continuing education, as well as the overall goals of the health care system. In this paper, some new types of uncertainty, which extend existing typologies, are identified and the contexts and strategies to deal with them are studied.

**Methods:**

We carried out a mixed-methods study, consisting of a qualitative and a quantitative phase. For the qualitative study, 128 residents reported critical incidents in their clinical practice and described how they coped with the uncertainty in the situation. Each critical incident was analyzed and the most salient situations, 45 in total, were retained. In the quantitative phase, a distinct group of 120 medical residents indicated for each of these situations whether they have been involved in the described situations and, if so, which coping strategy they applied. The analysis examines the relation between characteristics of the situation and the coping strategies.

**Results:**

From the qualitative study, a new typology of uncertainty was derived which distinguishes between technical, conceptual, communicational, systemic, and ethical uncertainty. The quantitative analysis showed that, independently of the type of uncertainty, critical incidents are most frequently resolved by consulting senior physicians (49 % overall), which underscores the importance of the hierarchical relationships in the hospital. The insights gained by this study are combined into an integrative model of uncertainty in medical residencies, which combines the type and perceived level of uncertainty, the strategies employed to deal with it, and context elements such as the actors present in the situation. The model considers the final resolution at each of three levels: the patient, the health system, and the physician’s personal level.

**Conclusions:**

This study gives insight into how medical residents make decisions under different types of uncertainty, giving account of the context in which the interactions take place and of the strategies used to resolve the incidents. These insights may guide the development of organizational policies that reduce uncertainty and stress in residents during their clinical training.

**Electronic supplementary material:**

The online version of this article (doi:10.1186/s12909-015-0459-2) contains supplementary material, which is available to authorized users.

## Background

Traditionally and from an outer perspective, it is commonly believed that physicians manage any clinical situation without doubts; however, everyday medical decisions are typically made under conditions of uncertainty. This is particularly the case for resident physicians, as they often operate under circumstances they are not yet familiar with and the skills required for an effective response to uncertainty are extremely broad and not well-defined [1]. As a result, residents may experience fear of causing errors, anxiety, stress, frustration, and insecurity. During his or her training, the physician tends to develop personal strategies that take the health and safety of the patient into account and which, in turn, affect his or her education and personal satisfaction, together with the goals at the institutional level [2–6].

Uncertainty is not unique to the field of medicine, it is also found in other disciplines such as economics, physics, education, and psychology. Morin [7] argues that knowledge is an uncertain adventure that permanently entails the risk of error, so that uncertainty is inherent in the cognitive act. In the medical field, some authors have proposed classifications of uncertainty (Table 1). In 1979, Light [8] considered five areas where medical students experience uncertainty: expectations of the professor, adequacy of knowledge, appropriate diagnosis, effective treatment, and patient satisfaction. Note that Light’s framework includes clinical reasoning when considering diagnosis and treatment. On the other hand, Beresford [9] explicitly considered uncertainty in a clinical context and distinguished three types: technical (i.e., lack of knowledge to understand the situation), conceptual (lack of skills to put acquired knowledge in practice), and personal (unknown expectations and difficult communication regarding another person). In his model, uncertainty (among other elements) shapes medical decision making, which in turn affects the ethical and professional commitment of the physicians. Importantly, his view implies that the ethical dimension is situated within the person, rather than in the ambiguity of the situation.

**Table 1:** Characteristics of previous uncertainty models

Author	Nature of the study	Classification	Categories of uncertainty	Comments
Light (1979) [8]	Based on clinical reasoning	Five areas where medical students experience uncertainty	Expectations of the professor	Includes clinical reasoning when considering diagnosis and treatment
			Adequacy of knowledge	
			Appropriate diagnosis	
			Effective treatment	
			Patient satisfaction	
Beresford (1991) [9]	Based on empirical observation and interviews	Three types of uncertainty	Technical: lack of knowledge to understand the situation	Uncertainty shapes medical decision making, which affects the ethical and professional commitment of the physicians
			Conceptual: lack of skills to put acquired knowledge in practice	
			Personal: unknown expectations and difficult communication regarding another person	The ethical dimension is situated within the person, rather than in the ambiguity of the situation
Farnan et al. (2008) [10]	Based on critical incident interviews	Six categories derived from Beresford’s three types	Procedural skills	The model is based on the trajectory followed by medical residents and specifies an ordered series of coping strategies
			Knowledge of indications	
			Care transitions	
			Diagnostic decision making Management conflict	
			Goals of care	
Han et al. (2011) [11]	Based on a conceptual framework	Taxonomic structure of uncertainty in three dimensions	The source of uncertainty	The source of uncertainty:
			The substantive issue that gives rise to the uncertainty	As a probability
			The locus of uncertainty	As ambiguity
				Due to complexity
				Substantive issues of uncertainty are broadly categorized in:
				Scientific
				Practical
				Personal
				The locus takes into account whether the uncertainty is situated in:
				Patient
				Clinician

In 2008, Farnan et al. [10] interviewed 42 medical residents from the general medicine service at the University of Chicago. Based upon the 18 critical incidents derived from these interviews, they qualified the domains in Beresford’s model by relating uncertainty to the following six categories: procedural skills and knowledge of indications (technical uncertainty by Beresford); care transitions, diagnostic decision making and management conflict (conceptual) and goals of care (personal). These authors further pointed to the strong hierarchical relations that typically exist among the health professionals in the hospital when they outlined the trajectory followed by medical residents when coping with medical uncertainty. This trajectory specifies an ordered series of coping strategies, which starts with the resident going back to the literature, continues with consulting peers, senior residents, a specialty fellow, and, as a last resort, the attending physician. If this process takes too long—the authors argue—it might result in delays of indicated care and, as such, negatively affect to the patient.

More recently, Han et al. [11] proposed a taxonomic structure of uncertainty in health care, which reconsiders the concept along three dimensions: (a) the source of uncertainty, (b) the substantive issue that gives rise to the uncertainty, and (c) the locus of uncertainty. With respect to the source, they distinguish between uncertainty as a probability (in particular, as probabilities are strictly between 0 and 1, they encompass uncertainty about the event), uncertainty as ambiguity (which can be understood as a lack of precision about the probability of an event), and uncertainty due to complexity (which refers, for example, to the probability of events being conditional on a large set of often unknown factors). Substantive issues are broadly categorized in scientific uncertainty (about diagnosis, prognosis, causality, treatment, etc.), practical uncertainty (associated with the system and process of care), and personal uncertainty (i.e., how the uncertainty affects the relations of the individual with other individuals and the system, one’s own values, etc.). Finally, the locus takes into account whether the uncertainty is situated in the patient and/or in the clinician. Contrary to Beresford [9] and Farnan et al. [10], Han et al. did not derive their taxonomy from empirical data, but they rather proposed a conceptual framework that proves useful to classify uncertainty situations encountered in clinical practice.

Instruments have been developed to evaluate uncertainty in different contexts. For example, Gerrity et al. [12] and Allison et al. [13] developed questionnaires to explore the attitudes of physicians to uncertainty. Greco and Roger [14] and Carleton et al. [15] proposed tools for the broader population in order to quantify intolerance to uncertainty. Importantly, none of the previous studies explicitly considered coping strategies used to resolve or diminish medical uncertainty.

The aim of this study is threefold: First, a typology of uncertainty is presented that extends the typology of Beresford [9]. Second, we identify strategies implemented by medical residents in regard to uncertainty in clinical practice; the latter is important as it may affect the process of situated learning [16] by improving the logistics of the educational system and by an increase of professional satisfaction in resident physicians. Third, the relation between types of uncertainty and coping strategies is examined and discussed.

## Methods

We used a mixed-methods approach for the current study. In the first phase, we applied a qualitative methodology to identify coping strategies applied by medical residents to deal with critical incidents and to develop a questionnaire to measure these strategies in typical uncertainty situations. In the second phase, the new questionnaire was applied and analyzed using quantitative methods.

### Qualitative phase

#### Sample

The sample consisted of residents who were enrolled, from March 2012 to February 2013, in the Unified Medical Specialties Program of the Faculty of Medicine at the National Autonomous University of Mexico (UNAM) and who received their education in either a private or a public hospital at Mexico City. Both hospitals were selected for convenience, because of the facilities they offered and the large number of engaged residents (across all specialties, 139 in the private and 547 in the public hospital). They differed strongly with respect to the material resources they have available (e.g., specific drugs, blood bank supplies, and certain specialized equipment are more frequently available in the private hospital). Within each hospital, residents were selected from the specialties of Internal Medicine, Obstetrics and Gynecology, Surgery, and Pediatrics (the latter in the public hospital only, as Pediatrics was not available as a specialty in the private hospital). The selected specialties generally have large numbers of registered residents and are considered representative across all medical and surgical specialties. In total, 259 residents eligible for participation were invited to take part in the study; 159 (61 %) effectively participated. However, 31 were excluded as their reports did not provide any useful information (because the residents typed random characters or comments unrelated to the topic into the response box or because they did not provide any answer at all). As a result, the reports of 128 residents were included in the qualitative analyses (64 % were affiliated with the public hospital and 36 % with the private hospital). Summary statistics for this sample are provided in Table 2. Note that the higher percentage of females in the second sample is due to the larger number of female residents in Family Medicine.

**Table 2:** Descriptive statistics for the demographic variables

		Qualitative phase (n=128)	Quantitative phase (n=120)
Year of residence	1	36 % (46)	45 % (54)
	2	25 % (32)	27.5 % (33)
	3	21 % (27)	27.5 % (33)
	4	18 % (23)	
Gender	Male	48 % (61)	28 % (33)
	Female	52 % (67)	72 % (86)
Age	<= 26	30 % (39)	26 % (31)
	27 - 29	59 % (75)	51 % (61)
	30 – 32	10 % (13)	18 % (22)
	>= 33	1 % (1)	2 % (2)

#### Technique for data collection: The critical incident

The information in the qualitative phase was collected through the technique of critical incidents. This is generally considered a resource that involves collecting detailed reports of instances or episodes in which a person does something especially effective or ineffective in order to achieve some goal [17]. Chell and Pittaway [18], for example, defined the critical incident as a qualitative interview procedure in order to understand an episode from the perspective of the individual who experienced it, taking into account cognitive, affective and behavioral elements. In the same vein, Soler [19] defined critical incidents as reports of introspection, that is, messages that the subject emits about his/her own behavior and experience. In the present study, the critical incidents allowed us to derive typical situations from the experiences of residents and relate them with the reported strategies used to cope with uncertainty.

#### Instrument

Participants were asked (a) to respond nine questions on personal and academic data (including year of residency, hospital location, medical specialty, sex, marital status, age, school of origin, and average score on the Mexican national entrance exam to resident education) and (b) to narrate an unexpected event in the last month of his/her medical practice that caused them uncertainty to deal with. With respect to the latter, the residents were asked to be specific about where, when, and how the event happened; whether or not someone else was present or help was looked for; the actions (taken by others or by themselves) they considered correct or incorrect and why (particularly whether or not the actions were according to the rules); the obstacles they encountered; the reasons why the event caused uncertainty (i.e., why they did not know how to deal with the problem); the patient’s condition and whether or not his or her safety was in jeopardy; the lessons learned from the incident and whether they were satisfied with their performance. Finally, two additional questions about the unexpected event were asked: the degree of uncertainty and the degree of stress it generated. Responses on the latter questions followed a Likert-type format, ranging from 1 (low) to 5 (very high). Although the survey allowed for the possibility to narrate a second critical incident, only one resident effectively did so. Hence, a total of 129 critical incidents were reported and analyzed.

#### Procedure

All residents eligible for participation received an e-mail with an invitation to respond to the questionnaire. The e-mail contained a hyperlink to a webpage and details on, amongst others, the time period that it would be active. The open source program LimeSurvey® 1.91 (https://www.limesurvey.org/en/) was used to collect the residents’ responses.

#### Analysis

For each critical incident, a document was derived that presented all information provided by the resident in a systematic way. As a first step in the analysis, five critical incidents were randomly selected and jointly revised by three raters (LH, TV, SG) to standardize the analytical procedure. In order to check the consistency among the three raters, five other critical incidents were selected and independently analyzed and the resulting classification was compared. Next, the 119 remaining critical incident reports were equally distributed among the three raters for further processing.

The procedure applied to each critical incident implied classifications according to type, to strategy used to confront it, and to the antecedents of the stress it generated. Subsequently, one or more typical situations were derived from each critical incident based on its general features, together with typical response options, that is, coping strategies applied to deal with the uncertainty described. Thus, the relationship between the properties of medical uncertainty and the coping strategies appears in the situations and responses, respectively. (See Additional file 1 for examples.)

In addition to the situations derived from the critical incident reports, and in order to enrich the questionnaire we aimed to construct, we also considered questions from other instruments [12–15]. Whenever possible, these questions (and situations derived from them) were classified according to the type of uncertainty specified in the original documents.

A content validation [20] of all collected situations was then performed, which led to a typology of uncertainty and a decision on which situations and responses to include in the final version of the questionnaire. Content validation was based on the relationship between the wordings of the situations on one hand and the constructs they attempted to measure on the other hand. Experts revised the classification of the situations according to type of uncertainty generated and further checked for repeated (or nearly identical) situations.

### Quantitative phase

#### Sample

A sample of 120 residents, who were receiving their training in Family Medicine in April 2013 at the Faculty of Medicine, were invited to participate in the second phase of this study. Table 2 shows the distribution of some demographic variables in the sample.

#### Instrument

The quantitative questionnaire is a product of the previously described qualitative phase and, hence, the details of this questionnaire are presented in the *Results* section.

#### Procedure

At the end of a regular class, the participants were invited to respond to the questionnaire. Responses were collected through pen-and-paper format. After all participants had completed the instrument, they were invited to a group session to share their opinion and comments on how the questionnaire could be further improved.

#### Analysis

The prevalence of each of the typical situations was analyzed by the frequency of the response “I have never been in that situation”. Furthermore, the frequency distribution of coping strategies across the situations was checked and it was examined how these frequency distributions depend on the type of uncertainty generated by the situations.

### Ethics

Ethical approval for this study, under the project named “Strategies of the resident physician to situations of uncertainty during critical incidents” (DGAPA-PAPIIT IN201514), was obtained from the Research and Ethical Board of UNAM Faculty of Medicine. In order to ensure anonymity, neither in the qualitative nor in the quantitative phase, personal information that might identify the participants was collected. The online format used to collect critical incidents on the qualitative phase, included a box with the following legend: “I accept voluntarily to participate in this study” that the residents had to mark and be able to proceed. In the quantitative phase where the questionnaire was applied to family medicine residents in person, they were asked verbally whether they agree to collaborate with their responses. Furthermore, the residents were informed that their participation would not affect their academic records.

## Results

### Qualitative phase

We describe three types of results obtained from the qualitative phase: (a) the typology of uncertainty derived from the critical incident reports, (b) the relation between the (type of) critical incidents and some context and perception variables derived from the reports, and (c) the final version of the questionnaire with selected typical situations and responses, which allows to quantitatively investigate the coping strategies employed by physicians faced with medical uncertainty.

#### Typology of uncertainty

Although we initially adopted the classification scheme of three types of uncertainty (technical, conceptual, personal) proposed by Beresford [9], our analysis of the critical incidents showed the need to modify and extend this classification. In particular, two new types of uncertainty arose, namely systemic and ethical uncertainty. We further renamed personal uncertainty as communicational uncertainty. The resulting typology distinguishes between five types of uncertainty, which are summarized with a short description in Table 3.

**Table 3:** Types of uncertainty

Type of uncertainty	Description
Technical	Lack of theoretical information resulting in ignorance for guiding actions
Communicational	Inability of the physician to communicate effectively and reach a joint decision with the patient
Conceptual	Inability to apply abstract knowledge in concrete situations
Systemic	Inability to act appropriately due to the lack of technological, technical, material and human resources as well as to ignore or act outside the standards and rules of the health system
Ethical	Inability to act when the person displays behaviors, attitudes and emotions inconsistent with the values and sociocultural codes of society, the institution and/or the person

#### The relation between critical incidents and context and perception variables

Across all 129 critical incidents, the resident explicitly reported in 9 % of the cases that patients were involved and in 14 % that the patient’s family was involved; peer residents played a significantly role in 17 % of the critical incidents, while senior residents and the responsible attending physician were present in 33 and 52 % of the cases, respectively; furthermore, other health professionals were involved in 40 and 5 % of the critical incidents concerned the institutional authorities. Logistic regression did not show significant differences between the presence of other health care personnel, patient or family on one hand, and the type of uncertainty elicited by the situation on the other hand, except for the case that communicational uncertainty was strongly related to interactions with the patient (*χ*^2^(4) = 18.0, *p* < .01) and his or her family (*χ*^2^(4) = 19.6, *p* < .01). One may note that the lack of significant results may be partly due to the small number of critical incidents associated with certain types of uncertainty.

In 30 % of the 129 critical incidents, the resident reported a positive learning experience from the situations. In 63 reports, the resident made a statement on his overall level of satisfaction of dealing with the situations, which in 62 % of the cases turned out to be positive. In 89 % of the critical incidents where the effect on the patient’s health was mentioned (115, in total), the outcome favored the patient. Finally, in 38 % of the critical incidents (24 of 63 reports), the resident indicated that the situation was resolved in accordance with the goals and rules of the system. Interestingly, the latter was found to be significantly more common in critical incidents classified under systemic uncertainty (viz., in 80 % of 40 reports, *χ*^2^(4) = 18.2, *p* < .01).

Table 4 shows the mean level (with standard deviation and 95 % confidence interval) of perceived uncertainty and perceived stress across the critical incidents for each of the five types of uncertainty (as measured on a five-point scale). First of all, the results show that stress and uncertainty are rather strongly correlated (*r* = .60, *p* < .01). Moreover, analyses of variance showed that mean levels of stress and uncertainty differ significantly among the five types of uncertainty (*F*(4,124) = 2.93, *p* = .02, for stress; *F*(4,124) = 2.80, *p* = .03, for uncertainty), with critical incidents that generate conceptual uncertainty leading to lower levels of stress and uncertainty.

**Table 4:** Number of critical incidents and levels of uncertainty and stress

Typology		Level of uncertainty			Level of stress		
	N	Mean	95 %-conf. int.	SD	Mean	95 %-conf. int.	SD
Technical	20	3.75	3.44, 4.05	.716	3.75	3.42, 4.05	.716
Conceptual	32	3.09	2.73, 3.48	1.058	3.28	2.91, 3.67	1.170
Communicational	18	3.89	3.54, 4.23	.758	3.94	3.53, 4.33	.873
Systemic	55	3.51	3.20, 3.80	1.153	3.95	3.66, 4.22	1.061
Ethical	4	4.25	4.00, 5.00	.500	4.50	4.00, 5.00	.577
Total	129	3.52	3.34, 3.69	1.039	3.77	3.59, 3.94	1.042

#### Questionnaire

The final version of the quantitative questionnaire derived from the critical incidents includes 45 typical situations which generate medical uncertainty. Each of these situations is associated with a particular type of uncertainty: six situations are assumed to generate technical uncertainty, five conceptual uncertainty, 11 communicational uncertainty, 15 systemic uncertainty, and eight ethical uncertainty. The situations are converted to items in the questionnaire by adding the question: “What did you do the last time that you found yourself in the following situation?” Each item has twelve response options (identical across all items), from which the respondents are asked to select one. The response option “I have never been in this situation” is one of the alternatives, while the other 11 comprise different coping strategies as a response to the uncertainty generated. The full set of 45 situations and 12 response options is included in Additional file 2.

### Quantitative phase

The main results of the 120 questionnaires applied to Family Medicine residents are shown in Table 5. The table presents the distribution of responses across all participants and all situations of a particular type. It was found that, across all combinations of residents and situations, 64 % of the responses indicated that the respondent had encountered him or herself in the situation. Not surprisingly, this percentage increases with the number of years in residence (49, 71, and 80 % for first-, second-, and third-year residents, respectively).

**Table 5:** Responses’ percentages according to type of uncertainty

	Typology of uncertainty					Total
Responses	Technical	Conceptual	Communicational	Systemic	Ethical	
I have not been in that situation.	40 %	32 %	38 %	23 %	47 %	36 %
Strategies						
I consulted with senior physicians.	65 %	53 %	53 %	35 %	42 %	49 %
I consulted with my peers or colleagues with a lower academic degree.	7 %	7 %	8 %	8 %	13 %	8 %
I consulted with non-medical personnel	2 %	1 %	2 %	6 %	4 %	3 %
I consulted with a medical committee.	3 %	4 %	3 %	8 %	7 %	5 %
I consulted with the patient or family.	0 %	5 %	2 %	1 %	0 %	2 %
I consulted informational sources (books, internet, etc.).	10 %	6 %	9 %	9 %	11 %	9 %
I followed the clinical guidelines.	5 %	7 %	6 %	13 %	7 %	8 %
I made my decision without consulting anyone.	5 %	7 %	11 %	14 %	9 %	9 %
I delegated the process of the incident.	2 %	3 %	3 %	3 %	4 %	3 %
I requested laboratory and imaging studies.	2 %	1 %	0 %	1 %	2 %	1 %
I followed a different strategy.	1 %	4 %	2 %	3 %	1 %	2 %

*Note: The percentages corresponding with each strategy are relative to the total number of situations the residents report to have experienced (see first row). Therefore, the percentages across rows (omitting the first) within each column sum 100 %

When a resident finds him or herself in a critical incident, the most common strategy used to resolve the uncertainty involves senior physicians (almost half of the cases), followed by informational sources (9 %), peers and residents of a lower rank (8 %), and sticking to the rules (8 %). Remarkably, in 9 % of the cases residents indicated they made a decision without consulting further sources. Interestingly, the coping strategy is significantly related to the type of uncertainty. As a meaningful pattern, we note that technical uncertainty (more than other types) is relatively more resolved by consulting senior residents (65 %) and informational sources (10 %), while for systemic uncertainty, it is relatively uncommon to consult a senior resident (35 %). In the latter case, sticking to the rules (13 %) or continuing without further consulting (14 %) are more frequent (as compared to the prevalence of these coping strategies for other types of uncertainty).

## Discussion

The project that led to the current study started as an exploration of how medical residents deal with uncertainty arising from critical incidents during their training. During the qualitative data analysis, we felt the need to reconsider the existing typologies on medical uncertainty; in particular, Beresford’s concepts turned out to be insufficient in order to cover the full range of uncertainty reported by the residents in our study. These considerations led us to propose an extended classification of uncertainty in clinical practice, which now includes institutional context (systemic) and dilemmas related to professionalism (ethical) in health care. The first two types of uncertainty (technical and conceptual) of the three proposed by Beresford were recovered [9], while the third (personal) was reformulated under the concept of communicational uncertainty. For systemic uncertainty, a variety of situations involved with the organizational culture, rules, resources and functioning of the hospital were found. As for ethical uncertainty, circumstances abounded where ruptures of regulations were tolerated and conflicts of interest were common. Beresford’s model was further extended by Farnan et al. [10] who identified six categories within the three types described by the former (see Table 1).

Our typology differs from Light [8] in that he conceived his categories from within the framework of clinical reasoning; however, we found that uncertainty in medical practice goes beyond biomedical logic and affects different levels of interaction in a context where the medical resident fulfills many roles [12]. Furthermore, our typology bears similarities with the taxonomy of Han et al. [11]: Conceptual and technical uncertainty basically correspond with “scientific uncertainty”, while systemic uncertainty can be considered equivalent to “practical uncertainty”, and communicational and ethical uncertainty coincides with Han et al.’s personal uncertainty. One may note, however, that our typology only considers the locus of the physician and, hence, it is situated within a part of the model by Han et al., who additionally consider the locus of the patient.

**Fig. 1:**
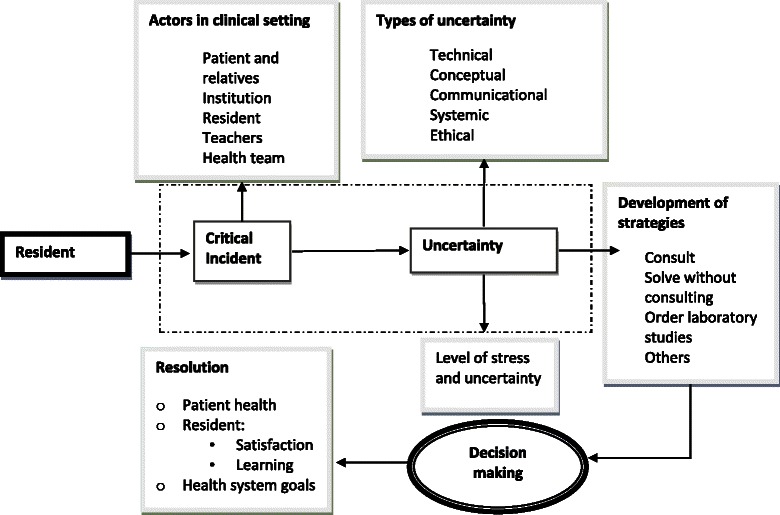
“Conceptual model of the different elements that interact when medical residents face uncertainty in clinical practice”. Uncertainty on critical incidents takes place within the clinical context and may be of different types, where distinct strategies may be activated to evaluate, decide and resolve the event. Stress and uncertainty may rise to different levels of intensity and the resolution may respond to diverse issues: patient health, resident’s learning and satisfaction, and health system goals

Based upon the critical incidents in the qualitative study, together with the responses obtained from the questionnaires in the quantitative phase, we built a conceptual model, which represents the process faced and the trajectory followed by a resident when he/she is confronted with an extraordinary clinical situation that generates uncertainty (see Fig. 1). This model is relevant because it includes the interactions with different actors implied in the situations, which generally affect the strategies, decisions and actions taken in the situation and, in turn, may intensify the stress and uncertainty experienced by the resident. The context in which these actions take place is explicitly taken into account by reference to human and material resources, the prevailing norms in the hospital, and the patient’s expectancies and necessities. Furthermore, the model incorporates the effects in relevant domains, including the patient’s health, the organization of the system, and the satisfaction and learning experience of the resident. We now discuss each of the components of the model in light of the qualitative and quantitative results in the present study and, where appropriate, relate them to results from similar studies on medical uncertainty.

Obviously, the first and central component of the model is the medical resident, who provided the information for this study as the narrator of the critical incidents and by responding the questionnaire, so he/she is the pivotal figure in the model; even though the supplied information is largely subjective, his or her point of view is central and provides a temporal and spatial structure for the residents’ experiences, which subsequently are disclosed through language [21]. The resident enters the scene as the principal character, through the interaction with other elements, the context characteristics and socio-cultural structures, he/she shapes a judgment of the situation, decides and acts upon it. How the resident relates to the clinical field affects his strategies to cope with uncertainty. For example, the more frequent and longer a resident has been exposed to extraordinary clinical situations, the more likely it is that he or she has experienced situations of uncertainty. Nevalainen et al. [22] found that more experienced general practitioners seem to better tolerate uncertainty and also seem to fear medical errors less than their young colleagues, which is similar to the findings of our study.

The other actors that show up in the critical incidents play their roles within strongly organized hierarchical structures. Indeed, the resident typically interacts with other members of the health team, including junior and senior residents, nurses, stretcher-bearers, the supervising physician, academic and administrative staff, amongst others. For obvious reasons, the resident’s relation with the patient and his or her family constitutes a key element in many critical incidents and turns out to be particularly correlated with communicational uncertainty. The importance of good communication skills in medical care and the ability of doctors to identify more accurately the problems of their patient, increment the likelihood that patients will be satisfied with their care and understand treatment options [23]. Some of the most common patient-physician communication deficiencies are: not allowing the patient to narrate the problem in his own words and interrupting him early, inability to bring out all the patient’s apprehensions, inability to appreciate the patient’s anxieties and worries, not assuring the patient that all his problems will be addressed, undermining the patient’s role in the decision making process, and not ensuring that the patient has understood the decision taken by the clinician on his/her behalf [24].

The conceptual model further considers the coping strategies developed by the resident as a response to the extraordinary situation he or she has been presented with. (Note that the list of coping strategies in the Figure is incomplete; the response options to the quantitative questionnaire represent a more extensive list, see Additional file 2). As stated before, the most frequent coping strategy adopted by a resident to respond to medical uncertainty consists of consulting senior residents or the responsible physician; as such, this result confirms the functionality of the hierarchically organized structure. In this respect, it is interesting to mention the study by Farnan et al. [10], who also report a hierarchical structure in strategies to cope with uncertainty. However, the nature of their hierarchy is quite distinct, as it defines a particular order among the coping strategies (going from consulting the literature to discussions with peers and senior residents and then seeing the attending physician). Our results do not provide evidence of such a sequential process; rather, the strategies to deal with uncertainty appear to operate simultaneously in a more holistic way and vary in function of the type of uncertainty elicited by the situation. Possibly, the contextual and organizational structure of the general medicine service at the University of Chicago versus the clinical services at the hospitals associated with UNAM’s Medical Specialties Program may explain the differences between both studies.

The uncertainty elicited by a critical incident may cause distinct degrees of perceived stress and uncertainty in the resident. Stress in physicians, particularly during residency training, has been an area of investigation for decades and is most often attributed to long working hours [25], sleep deprivation [26], the high number of patients they need to take care of, an overload of information and high responsibilities, the practical organization of the medical services and relatively low salaries [27], as well as the constant rotation among services [28]. Our study adds to this list of stress factors the uncertainty and acute stress following critical incidents. As a general rule, stress decreases if the resident can fall back on support networks, if his or her team members show a strong commitment, if sufficient material and human resources are assigned to the team, and if the learning experience associated with his or her resident education is generally positive [29].

The last component of the model presented in the Figure refers to the final resolution of the critical incident, which is considered from three perspectives: (a) how the critical incident affected the care and health of the patient, (b) how it contributed to the learning experience and satisfaction of the resident, and (c) to what extent the overall goals of the system are accomplished. The information in the reports shows that in the large majority of cases (about 80 %), the way the critical incident was resolved positively affected the patient’s health. This result may point to a generally efficient response to critical incidents in medical practice. The resident’s own learning experience and the satisfaction with how the critical incident was resolved is seen by the residents somewhat less favorably (60 % consider the learning experience positive and 30 % was satisfied with the solution); however, according to experiential learning theory [30], uncertainty situations constitute a crucial element in the development of professional skills. The resolution of a critical incident conform to the goals of the system may be difficult to accomplish, considering that the exceptional character of the event often acts upon and changes the hierarchical orders that are typical of the system, which in turn increases the uncertainty and which needs to be reestablished using the appropriate coping strategies.

The findings of our study suggest that the different components in our model are strongly interrelated. The strategies used by medical residents to cope with critical incidents depend on the type of uncertainty and the presence of health professionals, patient, and family, which further affect the levels of perceived stress and uncertainty. The type of uncertainty is also conceptually related to the resolution of the critical incident. The institutional context in which the resident works explains in part the level of stress reported in situations of systemic uncertainty, which may be due to the exposure of a prolonged stressful environment where residents are familiar with hospital standards but experience continual pressure in their work activities. Communication is an area often neglected in medical training, resulting in poor skills by residents to face situations involving patient-physician relationships, which is expressed in the high level of uncertainty. When residents had general abstract notions on how to resolve the situation (as in the case of conceptual uncertainty, see the definition in Table 3), there was less uncertainty about the way to proceed, which explains the difference between both types of uncertainty. Meanwhile, ethics is an issue not deeply considered by residents in clinical practice, with a tendency to hide it from open discussion; ethical issues are preferably discussed face to face.

The extensive qualitative part of the study, which implied the collection and analysis of the critical incidents in the first phase of the current project, led to a set of questions that were combined in an instrument. The instrument allowed examination of the strategies used by medical residents to cope with different types of uncertainty. The qualitative methodology to validate the questionnaire draws upon Stake [31] and consists of, among others, cross validating its applicability in other institutional contexts; in this case, the residents from Family Medicine who responded the pilot questionnaire. The latter part of the study allowed us (a) to comprehensively take account of the complex contextual nature of the phenomenon of uncertainty in medical practice, (b) to verify the correct interpretation of the uncertainty situations presented as different cases, (c) to adjust the response options, and (d) to fine-tune intermediate versions of the instrument by analyzing and incorporating the observations provided by the residents. Furthermore, the group discussions with experts, including the sessions where we debated the extended typology, added towards the validation of the instrument. In particular, the repeated discussions and revisions of the questionnaire allowed us to differentiate further our proposed typology of uncertainty from existing models, and helped confirm the new classification. Indeed, the theoretical comparison with previous models, the observations from the residents who are at the heart of uncertainty in medical practice, as well as the expert views on the topic, allow us to conclude that the instrument can be considered qualitatively validated. Based on data collected in a follow-up study with a large group of residents, the authors are now preparing another paper which offers quantitative evidence of the instrument.

As a final note, we highlight that the 45-item questionnaire derived from the critical incidents has an interesting feature which makes it different from the measurement instruments proposed by Greco and Roger [14] and Carleton et al. [15]: the respondents report their actual responses in situations they experienced in reality (“What did you do last time…?”), contrary to responses to hypothetical situations (“How would you react if…?”). One may note that the qualitative studies by Beresford [9] and Farnan et al. [10] use written or oral reports of critical incidents experienced in reality and, as such, are similar to the present study. With respect to responses to hypothetical versus real situations, a vast array of studies in the area of social psychology has been published on the gap between intentions and behavior [32–34]. Although most psychological theories view intentions as a direct precursor of behavior (like in the theory of reasoned action [35] or the theory of planned behavior [36]) and relatively high correlations have been found that allow to predict behavior from intentions, it is generally acknowledged that other factors restrain this association. As a result, it seems safe to assume that our approach yields results that resemble closely how residents deal with uncertainty in professional situations (although direct observational studies may contribute additional evidence about the behavior of residents with respect to uncertainty).

Among the most important limitations of this study we consider the rather small sample in the quantitative phase and the fact that the 120 residents surveyed represented a single specialty (Family Medicine). Residents of other specialties may respond differently. In the same vein, our qualitative study included only residents from two specific hospitals and some of the findings may reflect or may be attributed to the specific circumstances in which the residents in our study had their resident education. Furthermore, dissimilarities between the public and private hospital were not analyzed because the aim of the study was to construct the uncertainty typology; elsewhere, we report the variability within the organizational culture in both kinds of hospitals [37]. Based upon the results of the present study, future work may design and evaluate policies that should be issued at the institutional level in order to guide the residents to manage critical incidents and uncertainty more efficiently and appropriately. Other suggestions for future research include gathering evidence for the extended typology, the proposed conceptual model, and the questionnaire derived based on the critical incident reports in other geo-cultural contexts and possibly involving other members of the health care team. As Han indicates [38] the need for greater conceptual clarity, and consistent representational methods that make the meaning of various uncertainties understandable are pending issues. Also important are clinical interventions to support patients in coping with uncertainty in decision making.

## Conclusions

In this study, the typology proposed by Beresford [9], which has been considered in multiple publications as an appropriate way to structure uncertainty in medical practice, was extended to incorporate systemic and ethical uncertainty. We propose a new holistic model that accounts not only for the logic and the viewpoint of the resident in making decisions under uncertainty, but also explicitly considers the context in which the critical incident needs to be resolved. The described model is focused on uncertainty in resident education and allows for the analysis of the residents’ clinical experiences in relation with the everyday work environment. Our study is, after the publication by Farnan et al. [10], the second that explores uncertainty and associated coping strategies in medical residents.

## Additional files

Additional file 1: **“Examples of Critical Incidents sort by different type of uncertainty”.** The file includes five examples of the critical incidents written by the residents to illustrate different types of uncertainty (technical, conceptual, communicational, systemic and ethical). (PDF 225 kb) Adittional file 2: **“Questionnaire applied to family medicine residents”.** The file content refers to: (1) instructions to answer the instrument; (2) socio demographic and academic information; (3) 45 uncertainty situations that the residents may have experienced with 12 multiple choise options for each one. (PDF 269 kb)

## Abbreviations

UNAM: Universidad Nacional Autónoma de México [National Autonomous University of Mexico]

## References

[1] Luther VP, Grandall SJ (2011). Ambiguity and uncertainty: Neglected elements of medical education curricula?. Acad Med.

[2] Jarrillo E, Granados A, Chapela MC (2000). Estudiantes de medicina, un estudio de caso. Argumentos.

[3] Norma Oficial Mexicana (NOM 234-SSA1-2003) Utilización de campos clínicos para ciclos clínicos e internado de pregrado. [http://www.salud.gob.mx/unidades/cdi/nom/234ssa103.html].

[4] Timmermans S, Angell A (2001). Evidence-based medicine, clinical uncertainty, and learning to doctor. J Health Soc Behav.

[5] Ghosh BA (2004). Dealing with medical uncertainty. Minn Med.

[6] Sánchez M, Hernández M, Sánchez M, Martínez AI (2014). Toma de decisiones en Medicina Incertidumbre y probabilidad. Informática Biomédica.

[7] Morin E (1999). Seven complex lessons in education for the future.

[8] Light D (1979). Uncertainty and control in professional training. J Health Soc Behav.

[9] Beresford EB (1991). Uncertainty and the shaping of medical decisions. Hastings Cent Rep.

[10] Farnan JM, Johnson JK, Meltzer DO, Humphrey HJ, Arora VM (2008). Resident uncertainty in clinical decision making and impact on patient care: a qualitative study. Qual Saf Health Care.

[11] Han PK, Klein WM, Arora NK (2011). Varieties of uncertainty in health care: a conceptual taxonomy. Med Decis Making.

[12] Gerrity MS, Earp JAL, DeVellis R, Light D (1992). Uncertainty and professional work perceptions of physicians in clinical practice. Am J Sociol.

[13] Allison JJ, Kiefe CI, Cook EF, Gerrity MS, Orav EJ, Centor R (1995). The association of physician attitudes about uncertainty and risk taking with resource use in a Medicare HMO. Med Decis Making.

[14] Greco V, Roger D (2001). Coping with uncertainty: the construction and validation of a new measure. Pers Individ Dif.

[15] Carleton RN, Norton MA, Asmundson GJ (2007). Fearing the unknown: a short version of the Intolerance of Uncertainty Scale. J Anxiety Disord.

[16] Abreu LF, Infante CB (2004). Medical education challenged by the learning society. Gac Med Mex.

[17] Siegel ER, Rapp BA, Lindberg DA. Evaluating the impact of MEDLINE using the Critical Incident Technique. Proc Annu Symp Comput Appl Med Care. 1991: 83–87. PMC2247500 1807723

[18] Chell E, Pittaway L (1998). A study of entrepreneurship in the restaurant and café industry: exploratory work using the critical incident technique as a methodology. Int J Hosp Manag.

[19] Soler C (2013). Los constructos en las investigaciones pedagógicas: cuantificación y tratamiento estadístico. Atenas.

[20] Dornan T, Mann K, Scherpbier A, Spencer J. Learning and teaching in workplaces. In Medical Education Theory and Practice. 1st edition. Edited by Elsevier. 2011:193 – 210.

[21] Hamui SL. Las narrativas del padecer: una ventana a la realidad social. Cuicuilco, 2011; 52:51–70. http://www.redalyc.org/pdf/351/35124304005.pdf Accessed 25 Jul 2014.

[22] Nevalainen M, Kuikka L, Pitkäla K (2014). Medical errors and uncertainty in primary healthcare: A comparative study of coping strategies among young and experienced GPs. Scand J Prim Health Care.

[23] Maguire P, Pitceathly C (2002). Key communication skills and how to acquire them. Br Med J.

[24] Tyagi A, Garudkar S, Gagare A, Thopte A (2005). Medical Uncertainty: Are we better off in era of evidence based medicine?. Int Journal Med Res Health Sci.

[25] McCall T (1988). The impact of long working hours on resident physicians. N Engl J Med.

[26] Deaconson TF, O’Hair DP, Levy MF, Lee M, Schueneman AL, Condon RE (1988). Sleep deprivation and resident performance. JAMA.

[27] Hillhouse JJ, Adler CM, Walters DN (2000). A simple model of stress, burnout and symptomatology in medical residents: a longitudinal study. Psychology, Health & Medicine.

[28] Glick S (1988). The impeding crisis in internal medicine training programs. Am J Med.

[29] Badger LW, Chesebro MJ, Hartman JA (1987). First year residency stress: sources and mediators. Fam Pract Res J.

[30] Lifshitz A, Graue E, Sánchez M, Durante I, Rivero O (2010). La teoría experiencial del aprendizaje. Aprender de la experiencia. Educación en las Residencias Médicas.

[31] Ajzen I, Brown TC, Carvajal F (2004). Explaining the discrepancy between intentions and actions: The case of hypothetical bias in contingent valuation. Pers Soc Psychol Bull.

[32] Kühberger A, Schulte-Mecklenbeck M, Perner J (2002). Framing decision: Hypothetical and real. Organ Behav Hum Decis Process.

[33] Sheeran P. Intention-behavior relations: A conceptual and empirical overview. Eur Rev Soc Psychol 2002; 1–36.

[34] Fishbein M, Ajzen I (1975). Belief, attitude, intention, and behavior: An introduction to theory and research.

[35] Ajzen I (1991). The theory of planned behavior. Organ Behav Hum Decis Process.

[36] Hamui SL, Vives VT, Gutiérrez BS, Castro RS, Lavalle MC, Sánchez MM (2014). Cultura organizacional y clima: el aprendizaje situado en las residencias médicas. Inv Ed Med.

[37] Stake RE (2006). Evaluación comprensiva y evaluación basada en estándares.

[38] Han PK (2012). Conceptual Methodological, and Ethical Problems in Communicating Uncertainty in Clinical Evidence. Med Care Res Rev.

