# Supplementary material for: A typology of uncertainty derived from an analysis of critical incidents in medical residents: A mixed methods study
Source: BMC Med Educ. 2015 Nov 4;15:198. doi: 10.1186/s12909-015-0459-2 (PMC4634904; doi:10.1186/s12909-015-0459-2)
Supplement: Additional file 1: — “Examples of Critical Incidents sort by different type of uncertainty”. The file includes five examples of the critical incidents written by the residents to illustrate different types of uncertainty (technical, conceptual, communicational, systemic and ethical). (PDF 225 kb) [file 12909_2015_459_MOESM1_ESM.pdf]

Additional file 1

Examples of Critical Incidents sorted by different type of uncertainty.

Critical incident documented by a surgical resident (listed as technical uncertainty)

<table>
<tr><td>ID = 58</td><td>Age = 27</td><td>Year of residency = R2</td><td>Hospital = Public</td><td>Specialty= Surgery</td><td>Sex = Male</td></tr>
<tr><td colspan="6">Patient was seen in the outpatient service. Patient had a history of severe acute pancreatitis with multiple debridements secondary to necrotic and infected pancreatic pseudocyst. Computed tomography reported pseudocyst communicating with facial planes. I carried out the consultation without the aid of a general practitioner or senior resident. I sought consultation with both but was unsuccessful. The patient was reviewed without demonstrating any situation that required emergency management and was instructed to return for medical follow-up. After the patient was discharged, I received a response from a medical superior suggesting management that was not possible for me to carry out; however, the senior resident did not know the best course of action for the patient. It was a difficult situation because I did not have sufficient knowledge in regard to the management of such a patient plus it was an uncommon finding. I do not think the patient was at risk; however, he was informed about symptoms that would require returning to the Emergency Service. This performance was unsatisfactory.</td></tr>
<tr><td colspan="2">Uncertainty Rating = 4</td><td colspan="3">Stress Rating = 4</td><td>Another CI = No</td></tr>
<tr><td colspan="6">Comments:
This was a medical situation arising from a complication. Uncertainty was technical and there was lack of system support. The stress was generated due to lack of knowledge and insufficient resources for satisfactory resolution. The persons involved were senior physicians. The CI was resolved adversely for the patient.
Main uncertainty: technical and systemic
Main Strategy: consulting health team and resolving without further consultation
Main type of stress: lack of knowledge and insufficient resources for resolution
Question (technical uncertainty):
When you were confronted with a condition for which you had insufficient knowledge and did not find sufficient support from other physicians, what did you do?</td></tr>
</table>

Critical incident documented by a pediatric resident (listed as conceptual uncertainty)

<table>
<tr><td>ID = 29</td><td>Age = 25</td><td>Year of residency = R1</td><td>Hospital = Public</td><td>Specialty = Pediatrics</td><td>Sex = Female</td></tr>
<tr><td colspan="6">The incident occurred on the medical floor of the hemato-oncology on-call care service 1 month ago. I was with another R1 when a 27-year-old female patient diagnosed with acute lymphoblastic leukemia undergoing chemotherapy with a poor prognosis had a significant metabolic imbalance along with clinical deterioration. I was doubtful, despite knowing how to deal with this, as these are common cases. However, knowing the critical condition of the patient caused me distress and difficulty focusing on solutions. I consulted with the R3 on the shift. We immediately consulted a book describing pediatric emergencies and attempted to correct the metabolic imbalance; however, the patient’s condition deteriorated. Four hours later there was a change of shift and we contacted the general practitioner who identified the case and made the decision to transfer the patient to the pediatric intensive care unit. I think fear was based on personal decision making because when there is the option to contact another colleague, such anxiety decreases. I think a person with more extensive knowledge and expertise must be available to advise a lower-level resident.</td></tr>
<tr><td colspan="2">Uncertainty Rating= 3</td><td colspan="2">Stress Rating= 4</td><td colspan="2">Another CI = No</td></tr>
<tr><td colspan="6">Comments:
This was a medical condition with complications. Uncertainty was conceptual and the strategy was consulting a senior resident followed by seeking informational sources. Stress was generated due to lack of experience. The persons involved were a general practitioner and residents of equal or higher level. The CI was favorably resolved. The resident assessed her personal experiences satisfactory due to the availability of consultation with a colleague of a higher level during decision making.
Main Uncertainty: conceptual
Main Strategy: consult with senior resident and informational sources
Main type of stress: lack of experience
Question (conceptual uncertainty):
When you encountered a patient in critical condition and stress interfered with your decision making, what did you do?</td></tr>
</table>

Critical incident documented by a surgical resident (listed as communicational uncertainty)

<table>
<tr><td>ID = 6</td><td>Age= 26</td><td>Year of residency= R2</td><td>Hospital= Private</td><td>Specialty = Surgery</td><td>Sex = Female</td></tr>
<tr><td colspan="6">During my first year of residency I was working in the emergency room and had to evaluate a patient with data of acute abdomen. Subsequently, I introduced the patient to the ER physician who commented that the condition was not an acute abdomen and that I was incorrect. He decided to administer morphine, which I argued to the contrary in accordance with what was found on physical examination. The ER physician decided to admit patient to the floor but the patient’s condition continued to deteriorate. The patient was assessed by the surgeon who decided to operate, finding an internal hernia with vascular compromise of the affected segment. Fortunately, the patient progressed favorably. In this case, I had to approach the surgeon to request an evaluation of the patient upon admission to the floor. At that time, the decision was made to operate.</td></tr>
<tr><td colspan="2">Uncertainty Rating= 4</td><td colspan="2">Stress Rating= 5</td><td colspan="2">Another CI = No</td></tr>
<tr><td colspan="6">Comments:
This was a medical situation arising from a medical emergency. It was a communicated uncertainty with the healthcare team and the strategy was to consult the specialist physician. Stress was generated due to uncovered errors or omissions. The persons involved were the emergency physician and the specialist. The CI was resolved favorably for the patient.
Main Uncertainty: communicated with the healthcare team
Main Strategy: consulting medical specialist
Main type of stress: uncovering errors or omissions (treatment disagreement)
Question (communicated uncertainty):
What did you do when you disagreed with a decision made by the treating physician?</td></tr>
</table>

Critical incident documented by an internal medicine resident (listed as systemic uncertainty)

<table>
<tr><td>ID = 68</td><td>Age = 27</td><td>Year of residency = R2</td><td>Hospital = Private</td><td>Specialty = Internal Medicine</td><td>Sex = Male</td></tr>
<tr><td colspan="6">I was on duty at the hospital and received a call from the nursing staff reporting a cancer patient in the terminal stage (with palliative measures) who was smoking in her room. I immediately went to check on her. I talked to her and I emphasized hospital safety regulations for her, the healthcare team and other hospital patients. The patient replied that she was about to die and that gave her the freedom to do whatever she wanted with "what remained of her life" and she was not willing to stop because smoking provided her with "comfort". After talking with the patient and with instructions to turn off her oxygen supply and monitor her vital signs, I contacted her treating physicians. I reported the situation and received support from both of the physicians as well as from the security and nursing personnel. An agreement was reached with the patient in the calmest manner possible. Facing this experience, if you do not act in a calm and cautious manner, the situation may result in serious health, safety and even legal problems.</td></tr>
<tr><td colspan="2">Uncertainty Rating = 4</td><td colspan="2">Stress Rating = 3</td><td colspan="2">Another CI= No</td></tr>
<tr><td colspan="6">Comments:
This was an unusual medical situation. Uncertainty was systemic, and the strategy to confront it occurred in two stages: the first was resolved without consultation and the second by consulting a physician. The stress was generated due to being a situation “out of the norm.” The persons involved were the general practitioner, nursing and non-medical team. The critical incident was resolved favorably for the patient and the resident evaluated his own experience by adhering to the goals of the system and having the ability to act calmly in order to communicate with the patient.
Main Uncertainty: systemic
Main Strategy: consult a physician and health team
Main source of stress: situation outside of institutional standards
Question (systemic uncertainty):
What did you do when a patient violated institutional standards and you were present?</td></tr>
</table>

Critical incident documented by a pediatric resident (listed as ethical uncertainty)

<table>
<tr><td>ID = 49</td><td>Age = 30</td><td>Year of residency = R3</td><td>Hospital = Public</td><td>Specialty = Pediatrics</td><td>Sex = Female</td></tr>
<tr><td colspan="6">This was the case of an infant with dysmorphic syndrome (holoprosencephaly, short neck, left anophthalmia, cleft lip and palate, bilateral maxilla-mandible joint fusion) with airway access difficult for intubation. The patient's mother was reluctant to agree to tracheostomy. The patient was admitted to the pediatric intensive care unit for postoperative monitoring of plastic encephalocele surgery with phase III intubated ventilation via fiber optic endoscopy in the operating room. During the shift, the patient presented with severe bronchospasm and suspected extubation due to the presence of desaturation that did not recover with positive pressure. The general physician decided upon conventional intubation and, after multiple attempts (~12), support is requested of the anesthesiologist who attempted intubation three times unsuccessfully. Pulmonology Department was requested to perform fiber optic intubation; however, the necessary equipment was not available during the weekend. Support was requested of the Neonatology Department but without successful intubation. The patient was continually maintained with laryngeal mask with mild saturation recovery. Tracheostomy was recommended to the mother who agreed. However, when soliciting support from the adult otolaryngology service, they reported that they do not perform tracheostomy in young children. Support was then requested from pediatric surgery; however, due to being a weekend, this service was unavailable. There was consultation with assistant directors who were unable to offer alternatives. The patient suffered respiratory arrest and was successfully resuscitated with advanced maneuvers using a series of compressions, but subsequently died due to lack of airway access. I think that the patient should not have been resuscitated due to the poor prognosis, but resuscitation was done according to the indication of the general practitioner and refusal in this situation was not possible. There was no support from hospital authorities. My opinion is that patients who are not able to be properly cared for must not be accepted to the hospital, eliminating the behavior of accepting all patients regardless of their condition.</td></tr>
<tr><td colspan="2">Uncertainty Rating = 5</td><td colspan="2">Stress Rating = 5</td><td colspan="2">Another CI = No</td></tr>
<tr><td colspan="6">Comments:
This was a medical situation arising from a complication. Uncertainty was systemic and ethical and the strategy was to consult other specialties. Stress was generated due to patient safety. The persons involved were physicians from other specialties, chief resident and the treating physician. The CI was resolved in an unfavorable manner for the patient, medically and ethically. The resident criticized the policy of non-repudiation of the hospital and the general practitioner's decision, which the resident must follow without question. The resident also criticized the lack of support from hospital authorities.
Main Uncertainty: systemic and ethical
Main Strategy: consulting other specialties
Main type of stress: patient safety
Question (systemic and ethical uncertainty):
When the treating physician suggested performing a specific procedure that you disagreed with, what did you do?
Ethical question (ethical uncertainty):
When caring for a patient, you realize that the hospital does not have sufficient resources to treat the patient, what do you do?</td></tr>
</table>
