# Supplementary material for: A typology of uncertainty derived from an analysis of critical incidents in medical residents: A mixed methods study
Source: BMC Med Educ. 2015 Nov 4;15:198. doi: 10.1186/s12909-015-0459-2 (PMC4634904; doi:10.1186/s12909-015-0459-2)
Supplement: Adittional file 2: — “Questionnaire applied to family medicine residents”. The file content refers to: (1) instructions to answer the instrument; (2) socio demographic and academic information; (3) 45 uncertainty situations that the residents may have experienced with 12 multiple choise options for each one. (PDF 269 kb) [file 12909_2015_459_MOESM2_ESM.pdf]

Additional file 2

Questionnaire applied to Family Medicine Residents

Choose only one option that is closest to your response

I. I consulted with senior physicians

II. I consulted with my peers or colleagues with a lower academic level

III. I consulted with non-physician personnel (nurses, administrative, etc.)

IV. I consulted with a medical committee

V. I consulted with the patient or family

VI. I consulted informational sources (books, internet, etc.)

VII. I followed the clinical guidelines

VIII. I decided without consulting

IX. I delegated the process of the incident

X. I requested laboratory and imaging studies

XI. I followed another strategy

XII. I have not been in that situation

The following questions start with "What did you do when..."

1. During a procedure, because of lack of practice, you made a mistake with serious consequences for the patient's health?

2. The patient experienced complications due to an error arising from your lack of knowledge?

3. You supervised a lower-level colleague and encountered a complication that your colleague did not know how to resolve?

4. You encountered a difficult patient and were unsure of what action to take?

5. You were responsible for a patient without an accurate diagnosis and as time passed the patient's condition deteriorated?

6. You had to face a legal medical case and did not know what to do?

7. An emergency was presented and you were unprepared due to lack of practice?

8. One of your patients required a procedure for which you did not have sufficient practice?

9. You faced a highly dangerous procedure even for your own safety?

10. An adverse factor prevented you from concluding a procedure?

11. You had to convince a patient to undergo a life-saving procedure and the patient did not accept it?

12. You encountered a patient in critical condition and stress blocked your decision making?

13. The attending physician complicated your awareness of health care events?

14. You had to give unexpected bad news to a patient and the patient's family and you were unprepared as to how to handle this situation?

15. The decision of the family was against the advice you suggested?

16. A problem in communication among physicians of different services imposed a sanction?

17. You were blamed for a medical situation by an angry patient without it being your responsibility?

18. Inadequate communication with the patient generated a legal problem?

19. You did not know how to explain a difficult situation to your patient?

20. You had difficulty in explaining to a relative the risks and complications of the patient?

21. Because of lack of communication among residents, you did not have the information necessary to respond to a patient?

22. A patient asked for information of the condition and you had doubts about how to express it?

23. In an emergency, the health team coordination was poor and the quality of care was affected?

24. The hospital policies affected patient care?

25. During a procedure you needed the supervision of a specialist physician and he/she was not present?

26. An urgent situation arose and you did not have the support of the medical staff?

27. A patient assaulted you or another member of the hospital staff?

28. There was no drug of choice for the patient's treatment?

29. You required the service of another medical specialty and did not receive it?

30. During the weekend shift, materials needed for proper medical practice were unavailable?

31. Physicians from other shifts made decisions contrary to those reported by you and caused harm to the patient?

32. A patient violated the rules of the institution and you were present?

33. In an emergency situation, you were unable to locate the family to make a vital decision?

34. In an emergency, a hospital service refused to cooperate?

35. In an emergency you required laboratory studies and the delivery time of these studies did not meet the needs of the situation?

36. You hesitated over your medical decisions and there was no supervision and feedback of your work?

37. Excessive work load affected the quality of your consultations?

38. The family asked not to resuscitate the patient but the treating physician did not follow this instruction?

39. In a situation you had to decide between the patient's safety and your own?

40. You were questioned for not doing your work?

41. You assumed the responsibility for the error of a senior physician?

42. A colleague claimed that you withheld information?

43. You were aware that your colleagues were performing inadequate medical procedures due to lack of knowledge?

44. A senior physician ordered you to assist during a procedure that you doubted was correct according to the safety of the patient?

45. You realized that an omission in your work resulted in consequences for the patient?
